# Fractional order effects on solitary waves and chaotic regimes in the mKdV Burgers equation

**DOI:** 10.1038/s41598-025-25340-6

**Published:** 2025-11-21

**Authors:** Md. Antajul Islam, Nasrin Nahar Rimu, Pinakee Dey

**Affiliations:** Department of Mathematics, Mawlana Bhashani Science and Technology University, Tangail, 1902 Bangladesh

**Keywords:** Shock-like solitons, Bifurcation structures, Phase portraits, Plasma physics, Chaotic behavior, Mathematics and computing, Physics

## Abstract

This paper examines the space–time fractional modified Korteweg-de Vries Burgers (mKdV-Burgers) equation to address nonlinear wave dynamics of the equation through the improved F-expansion representation with the Riccati equation. The given strategy offers a methodical system of obtaining a wide category of precise analytical solutions, solitary wave solutions, kink-type solutions, periodic solutions, and rational solutions. The resulting results show the existence of dissipative and shock-like solitons, which add to the knowledge of nonlinear propagation phenomena in complicated media. Moreover, a dynamical study is performed in terms of bifurcation structures, phase portraits, Lyapunov exponents, and sensitivity analysis of changes between stable and chaotic states. These studies show that parameters of fractional order affect the stability and complexity of the system. This dynamical and analytical set of methods does not only confirm the efficiency of the improved F-expansion method but also contributes to new physical understanding of the fractional nonlinear evolution equations (FNLEEs). Findings may be generalized to fluid dynamics, plasma physics, and nonlinear optics, and the framework can be generalized to higher-dimensional or coupled fractional systems to control and predict multi-stable and chaotic behavior.

## Introduction

Nonlinear evolution equations (NLEEs) are used to model various and complex physical processes in fluid mechanics, plasma physics, optical fibers, biology, and engineered systems. The fundamental nonlinear interaction between dispersion, dissipation, and convection is described by these equations, resulting in the occurrence of solitons, shock waves, rogue waves, and turbulence^1–3^. An example is solitary waves in shallow water^4^, envelope solitons in plasma, and shock structures in aerodynamics, which are well-applicable within the scope of NLEEs. The second important hybrid NLEE is the so-called modified Korteweg-de Vries-Burgers (mKdV-Burgers) equation that includes nonlinear convective and nonlinear dispersion effects and forms a powerful mathematical model of nonlinear wave propagation in dispersive-dissipative media^5–8^. This equation as a fraction allows a more comprehensive study of the effects of memory, anomalous diffusion, and non-local interactions needed to assure a precise model of complex dynamical processes in physical systems.

Fractional calculus, which generalizes integer-order differentiation to non-integer orders, has proven highly effective for describing hereditary and nonlocal properties in various materials and media. It introduces fractional derivatives such as those of Riemann–Liouville^9^, Caputo^10^, and conformable^11^, each with distinct properties that generalize classical calculus. The $\beta -$ fractional derivative^12^ of the function $S\left( t \right)$ is given on the interval $t \in \left[ {0, \infty } \right)$:

$$D_{t}^{\beta } S\left( t \right) = \mathop {\lim }\limits_{h \to 0} \frac{{S\left( {t + \delta \left( {t + \frac{1}{{{\Gamma }\beta }}} \right)^{1 - \beta } } \right) - S\left( t \right)}}{h},\;{\text{where}}\; \, 0 < \beta \le 1.$$

Here $\beta$ is the fractional order, and $\Gamma \left( \beta \right)$ is the Gamma function. In this work, the $\beta -$ fractional derivative technique has been used to assess soliton dynamics, chaotic behavior, bifurcation processes, stability concepts, chaos sensitivity, Lyapunov stability, and dependence on parameters in fractional-order nonlinear evolution equations (FNLEEs). We search in the space–time fractional mKdV-Burgers Eq. (essential to define nonlinear wave propagation in the context of fractional power). This offers an integrated method of depicting wave interactions that preserve non-local and memory effects in complex systems.

The generalized mKdV-Burgers Eq. reads in its space–time fractional form as

1 $$D_{t}^{\beta } S + aS^{2} D_{x}^{\alpha } S + bSD_{x}^{\alpha } S + cD_{x}^{3\alpha } S = 0.$$

in which $D_{t}^{\beta } S$ is the profile of the wave, $aS^{2} D_{x}^{\alpha } S$ and $bSD_{x}^{\alpha } S$ are the higher-order and lower-order nonlinear convection terms, and $cD_{x}^{3\alpha } S$ is the dispersion effect. The fractional derivative introduction extends the classical model and enables a more precise description of dissipative-dispersive processes of unsteady wave propagation. These types of fractional models are extensively applied in fluid mechanics, rheology, control theory, and viscoelastic systems where memory and spatial heterogeneity are important in determining system behavior^13–16^. The use of hybrid algorithms and intelligent computational methods in other fields^17^ demonstrates the potential for extending our analytical study of fractional mKdV–Burgers equations to automated simulations or optimization of nonlinear wave behaviors.

The main objective of this work is to derive exact traveling wave solutions and perform an in-depth dynamical analysis of the space–time fractional mKdV–Burgers equation. To this end, the improved F-expansion method, based on the Riccati equation, is employed. This approach provides a systematic framework for constructing multiple solution families, including solitary waves, kink-type structures, periodic waves, and rational solutions. Compared to traditional methods, the improved F-expansion approach^18^ significantly broadens the solution space by incorporating more general polynomial structures, thereby enabling the generation of diverse nonlinear waveforms within a unified analytical setting.

In addition to being able to get precise solutions, this analysis conducts a detailed dynamical study with Lyapunov exponents, bifurcation structures, phase portraits, Poincare maps, and time series maps to study the transitions between periodic, quasi-periodic, and chaotic regimes^19–21^. This type of analysis can be useful in understanding the impact of fractional parameters on stability and the development of chaotic behaviors.

In recent decades, many different analytical approaches have been used to find exact and approximate solutions to NLEEs. They are the $\left( {G^{\prime} /G} \right)$—expansion technique^22–26^, the $\left( {G^{\prime} /G, 1/G} \right)$—expansion technique^12,27–30^, extended tanh function method^31,32^, the sine–cosine method^33^, Jacobi elliptic functions methods^34–36^, the Hirota bilinear method^37–39^, Darboux and Backlund transformations^40,41^, the Kudryashov method^42,43^, the Exp-function method^44,45^, and Lie symmetry^46,47^. Despite their effectiveness, many of these methods have limitations in handling fractional-order systems or mixed dispersive dissipative effects. The improved F-expansion method addresses these challenges by providing greater flexibility and computational efficiency, making it particularly suitable for fractional nonlinear equations that exhibit both localized and periodic nonlinear structures. Recent advances in fractional nonlinear evolution equations have revealed new insights into wave propagation, front dynamics, and chaotic transitions across diverse physical systems. Several related studies have enriched this field ranging from fractional Kolmogorov–Petrovskii–Piskunov and Fisher-type equations^48,49^, Heisenberg spin models^50^, and diffusion–reaction systems^51–55^, to optical and field-theoretic frameworks such as the fractional $\phi^{{4}}$, Landau–Ginzburg–Higgs, and Cahn–Allen equations^56–60^. These works collectively highlight how fractional calculus captures nonlocality, memory, and dissipation in soliton dynamics, and demonstrate the power of algebraic and extended direct methods for constructing exact wave solutions. Building upon these developments, the present study investigates the fractional-order effects on solitary waves and chaotic regimes in the mKdV–Burgers equation, employing an improved F-expansion method combined with dynamical system analysis to bridge analytical precision with nonlinear stability and chaos characterization. Although the present study focuses on the one-dimensional constant coefficient case, it establishes a theoretical foundation for potential extensions to higher-dimensional, variable-coefficient, and stochastic fractional systems. By integrating analytical, numerical, and dynamical approaches, this work not only enriches the theoretical understanding of fractional nonlinear wave equations but also provides practical insights into real-world dispersive–dissipative wave phenomena. In summary, the present study proposes a unified framework to analyze the space–time fractional mKdV–Burgers equation, combining exact analytical derivations with nonlinear dynamical analysis to deepen our comprehension of fractional nonlinear dynamics and chaotic wave interactions.

The paper is structured as follows: thirteen sections. The methodology procedure setting is defined in Sect. “Methodological procedure setting”, in which the method of the improved F-expansion has been used. In Sect. “Theoretical estimation and resolutions”, we demonstrate a theoretical estimation and resolutions by reducing the fractional PDE to an ODE to find solutions. Sect. “Impact of stretching exponents” covers the impact of stretching exponents on the soliton’s solutions, while Sect. “Physical significance of soliton” discusses the physical significance of soliton of these solutions. The qualitative dynamics of the system discussed in Sects. “Investigation of stability”, “Bifurcation”, “Chaotic nature”, “Multi-stability” and “Lyapunov stability” comprise stability, bifurcations, chaos, and multi-stability, as well as the analysis of Lyapunov stability. Sect. “Perspective research” provides research directions for the future, and Sect. “Evaluation of novelty” assesses novelty or compares the contributions with other literature. Lastly, Sect. “Conclusion” provides the conclusion of the study by mentioning the important findings and recommendations.

## Methodological procedure setting

The following part examines the systematic approach for acquiring precise soliton solutions to FNLEEs. Initially, we commence with a general nonlinear evolution model articulated as

2 $$R\left( {S,S_{t} ,S_{x} ,D_{t}^{\beta } S,D_{t}^{\alpha } S \ldots } \right) = 0,$$

where $S = S\left( {x_{i} ,t} \right);\left( {i = 1,2,3,4, \ldots } \right)$ denotes the spatio-temporal wave function, and the model may incorporate fractional derivatives of $S\left( {x_{i} ,t} \right)$. To diminish one dimensionality, we implement a wave transformation and derive a nonlinear ODE. The employed transformation is

3 $$S\left( {x,t} \right) = S\left( \xi \right).$$

Alongside $\xi = \frac{k}{\alpha }\left( {x + \frac{1}{{{\Gamma }\alpha }}} \right)^{\alpha } - \frac{\omega }{\beta }\left( {t + \frac{1}{{{\Gamma }\beta }}} \right)^{\beta }$, $\omega$ represents the wave speed. The associated ordinary differential Eq. is expressed as

4 $$R\left( {S,S^{\prime},S^{\prime\prime},S^{\prime}, \ldots } \right) = 0.$$

where the prime signifies the order of the differential Eq. pertaining to the new wave function inside its domain $\xi$. Like other analytical methods addressing the issue, the enhanced $F -$ expansion technique examines a series-type solution to the converted ODE. The planar solution to this Eq. is provided by

5 $$S\left( \xi \right) = \mathop \sum \limits_{n = 0}^{M} h_{n} \left( {m + F\left( \xi \right)} \right)^{n} + \mathop \sum \limits_{n = 1}^{M} r_{n} \left( {m + F\left( \xi \right)} \right)^{ - n} .$$

The answer integrates multiple arbitrary constants $h_{n}$, $r_{n}$, $m$, and a function $F\left( \xi \right)$, which is derived as a complete solution to the Riccati problem.

6 $$F^{\prime}\left( \xi \right) = p + F^{2} \left( \xi \right).$$

The Riccati Eq. yields multiple generic solutions dependent on the defined conditions for the free parameter $p$. The expansion in (5) can be regarded as a rational expansion of the solution to the Riccati problem (6), so enabling the use of the transformed rational function approach^61,62^. The Riccati Eq. yields many generic solutions contingent upon the value of the parameter $p$. The enhanced F-expansion approach utilizes Riccati Eq. (6) as an auxiliary relationship. This is justified by the Eqs. diverse solution families (hyperbolic, trigonometric, rational), which map to solitary, periodic, and kink-like structures in nonlinear dispersive-dissipative equations, facilitating order reduction in fractional mKdV Burgers traveling wave transformations. The Riccati model also aligns with physical expectations of localized solitary pulses and periodic oscillations in nonlinear wave propagation. Furthermore, the Cole-Hopf transformation connects the Riccati Eq. to a second-order linear ODE, establishing a link between nonlinear and linear structures in fractional-order systems.

**Category 1**: For $p \ne 0$, Eq. (6) represented a solution as

7 $$F\left( \xi \right) = \left\{ {\begin{array}{*{20}c} {\left\{ {\begin{array}{*{20}c} { - \sqrt { - p} \tanh \left( {\sqrt { - p} \xi } \right),p < 0} \\ { - \sqrt { - p} \coth \left( {p\xi } \right),p < 0} \\ \end{array} } \right.} \\ {\left\{ {\begin{array}{*{20}c} {\sqrt p \tan \left( {\sqrt p \xi } \right),p > 0} \\ { - \sqrt p \cot \left( {\sqrt p \xi } \right),p > 0} \\ \end{array} } \right.} \\ \end{array} } \right.$$

**Category 2**: For $p = 0$ , Eq. (6) signifies a solution as

8 $$F\left( \xi \right) = - \frac{1}{\xi }.$$

Currently, the balance law is being applied to identify the main nonlinear terms and the largest portion of the term involving linear dynamics in (3) as this will be of importance to obtain an estimate on the parameter $p$. A second change is included when $p$ is not a positive integer:

9 $$S\left( \xi \right) = w\left( \xi \right)^{N} .$$

After substituting (5) or (9) into ODE, after differentiating the above relation, the application of (6) gets a polynomial Eq. of $F\left( \xi \right)$ Setting the coefficients of each power of $F^{j} \left( \xi \right)$ and $F\left( \xi \right)$ to zero results in a system of algebraic Eqs. The unknown parameters are ascertained by resolving this system. Ultimately, this parameter value is substituted back into the proposed solution, yielding the sought-after accurate analytical solutions.

Limitations: The described approach is primarily effective for hyperbolic and trigonometric exact solutions, with limitations in representing complex structures such as rogue waves or breathers in fractional models. Although bifurcation theory and Lyapunov exponents were employed to investigate chaotic dynamics and stability limits, a complete understanding necessitates further dynamical system studies beyond the Riccati framework.

## Theoretical estimation and resolutions

Figure 1 shown as,

**Fig. 1:**
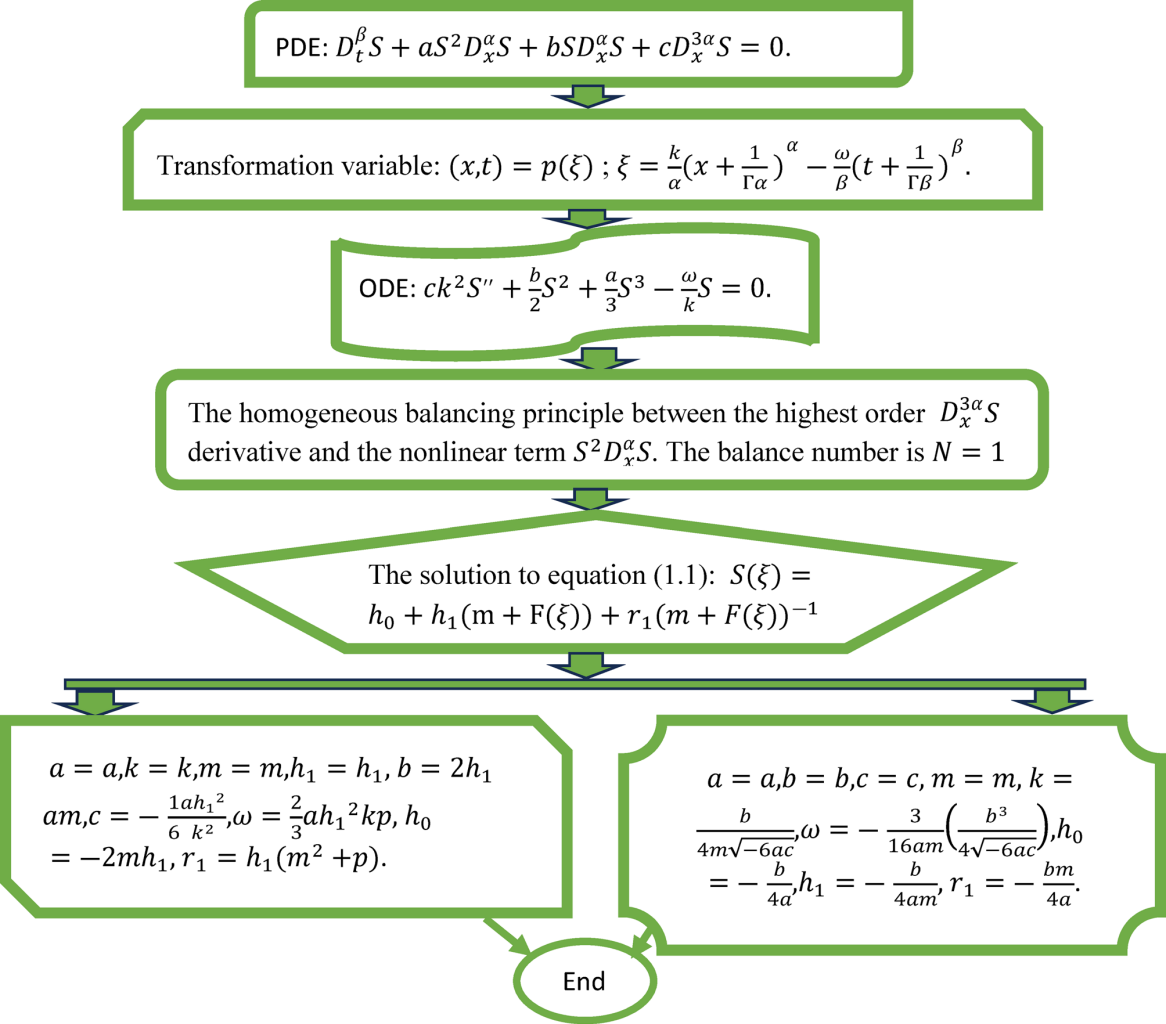
Flowchart of the theoretical estimation and resolution process.

In the hyperbolic and trigonometric solutions, the free parameters are: $a, k,m,h_{1} ,$ and $p$. The conditions are $a \ne 0$ (not to get a trivial equation) and $k \ne 0$ (not to divide the equation by zero in the coefficient $c = - \frac{1}{6}\frac{{ah_{1}^{2} }}{{k^{2} }}$). The free parameters in the rational solution are $a,b,c, \& m$.These conditions are $a \ne 0$ and $m \ne 0$ (because this case would have a trivial or undefined square root), $c \ne 0$ (to prevent division by zero in $k = \frac{b}{{4m\sqrt { - 6ac} }}$), and $- 6ac > 0$ (to ensure the square root is real) and $b \ne 0$ (to avoid trivial solution).

Both reported solutions have been systematically verified by substitution into the governing fractional PDE (1). To do this we used symbolic computation with Mathematica and Maple, which can simplify nonlinear fractional terms automatically under the $\beta$-derivative framework. In addition, we numerically verified the results in a few cases by plotting the residual error (i.e., the difference between the LHS and RHS of Eq. 1) over a spatial–temporal grid, confirming that the residuals were small, close to machine precision. This combined symbolic numerical validation provides strong assurance of the correctness of our solutions.

A generic solution including both hyperbolic and trigonometric functions of the Riccati problem is derived when $p \ne 0$. Consequently, upon substituting the aforementioned parameter values into the solution, we obtain the hyperbolic solution for the situation $p < 0$, expressed as

10 $$w_{1} \left( \xi \right) = - 2mh_{1} + h_{1} \left( {m - \sqrt { - p} \tanh \left( {\sqrt { - p} \xi } \right)} \right) + \left. {\frac{{h_{1} \left( {m^{2} + p} \right)}}{{m - \sqrt { - p} \tanh \left( {\sqrt { - p} \xi } \right))}}} \right).$$

and

11 $$w_{2} \left( \xi \right) = - 2mh_{1} + h_{1} \left( {m - \sqrt { - p} \coth \left( {\sqrt { - p} \xi } \right)} \right) + \left. {\frac{{h_{1} \left( {m^{2} + p} \right)}}{{m - \sqrt { - p} \coth \left( {\sqrt { - p} \xi } \right))}}} \right),$$

where $$\xi = \frac{k}{\alpha }\left( {x + \frac{1}{{{\Gamma }\alpha }}} \right)^{\alpha } - \frac{\omega }{\beta }\left( {t + \frac{1}{{{\Gamma }\beta }}} \right)^{\beta } .$$

The relevant solutions based on the governing model are then derived through the wave map and articulated in the following format.

12 $$\begin{aligned} S_{1} \left( {x,t} \right) = & - 2mh_{1} + h_{1} \left( {m - \sqrt { - p} \tanh \left( {\sqrt { - p} \left( {\frac{k}{\alpha }\left( {x + \frac{1}{{{\Gamma }\alpha }}} \right)^{\alpha } - \frac{{\frac{2}{3}ah_{1}^{2} kp}}{\beta }\left( {t + \frac{1}{{{\Gamma }\beta }}} \right)^{\beta } } \right)} \right)} \right) \\ & + \frac{{h_{1} \left( {m^{2} + p} \right)}}{{m - \sqrt { - p} \tanh \left( {\sqrt { - p} \left( {\frac{k}{\alpha }\left( {x + \frac{1}{{{\Gamma }\alpha }}} \right)^{\alpha } - \frac{{\frac{2}{3}ah_{1}^{2} kp}}{\beta }\left( {t + \frac{1}{{{\Gamma }\beta }}} \right)^{\beta } } \right)} \right)}}. \\ \end{aligned}$$

and

13 $$\begin{aligned} S_{2} \left( {x,t} \right) = & - 2mh_{1} + h_{1} \left( {m - \sqrt { - p} \coth \left( {\sqrt { - p} \left( {\frac{k}{\alpha }\left( {x + \frac{1}{{{\Gamma }\alpha }}} \right)^{\alpha } - \frac{{\frac{2}{3}ah_{1}^{2} kp}}{\beta }\left( {t + \frac{1}{{{\Gamma }\beta }}} \right)^{\beta } } \right)} \right)} \right) \\ & + \frac{{h_{1} \left( {m^{2} + p} \right)}}{{m - \sqrt { - p} \coth \left( {\sqrt { - p} \left( {\frac{k}{\alpha }\left( {x + \frac{1}{{{\Gamma }\alpha }}} \right)^{\alpha } - \frac{{\frac{2}{3}ah_{1}^{2} kp}}{\beta }\left( {t + \frac{1}{{{\Gamma }\beta }}} \right)^{\beta } } \right)} \right)}}. \\ \end{aligned}$$

Since here $N = 1$. Conversely, by presuming $p > 0$, the solution would be expressed in trigonometric form as

14 $$\begin{aligned} S_{3} \left( {x,t} \right) = & - 2mh_{1} + h_{1} \left( {m + \sqrt p \tan \left( {\sqrt p \left( {\frac{k}{\alpha }\left( {x + \frac{1}{{{\Gamma }\alpha }}} \right)^{\alpha } - \frac{{\frac{2}{3}ah_{1}^{2} kp}}{\beta }\left( {t + \frac{1}{{{\Gamma }\beta }}} \right)^{\beta } } \right)} \right)} \right) \\ & + \frac{{h_{1} \left( {m^{2} + p} \right)}}{{m + \sqrt p \tan \left( {\sqrt p \left( {\frac{k}{\alpha }\left( {x + \frac{1}{{{\Gamma }\alpha }}} \right)^{\alpha } - \frac{{\frac{2}{3}ah_{1}^{2} kp}}{\beta }\left( {t + \frac{1}{{{\Gamma }\beta }}} \right)^{\beta } } \right)} \right)}}. \\ \end{aligned}$$

and

15 $$\begin{aligned} S_{4} \left( {x,t} \right) = & - 2mh_{1} + h_{1} \left( {m - \sqrt p \cot \left( {\sqrt p \left( {\frac{k}{\alpha }\left( {x + \frac{1}{{{\Gamma }\alpha }}} \right)^{\alpha } - \frac{{\frac{2}{3}ah_{1}^{2} kp}}{\beta }\left( {t + \frac{1}{{{\Gamma }\beta }}} \right)^{\beta } } \right)} \right)} \right) \\ & + \frac{{h_{1} \left( {m^{2} + p} \right)}}{{m - \sqrt p \coth \left( {\sqrt p \left( {\frac{k}{\alpha }\left( {x + \frac{1}{{{\Gamma }\alpha }}} \right)^{\alpha } - \frac{{\frac{2}{3}ah_{1}^{2} kp}}{\beta }\left( {t + \frac{1}{{{\Gamma }\beta }}} \right)^{\beta } } \right)} \right)}}. \\ \end{aligned}$$

Finally, in the limit where $p = 0$, the solutions of the underlying model are rational, given by

16 $$\begin{aligned} S_{5} \left( {x,t} \right) = & - \frac{b}{4a} - \frac{b}{4am}\left( {m - \frac{1}{{\frac{{\left( {\frac{b}{{4m\sqrt { - 6ac} }}} \right)}}{\alpha }\left( {x + \frac{1}{{{\Gamma }\alpha }}} \right)^{\alpha } - \frac{{ - \frac{3}{16am}\left( {\frac{{b^{3} }}{{4\sqrt { - 6ac} }}} \right)}}{\beta }\left( {t + \frac{1}{{{\Gamma }\beta }}} \right)^{\beta } }}} \right) \\ & - \frac{bm}{{4a\left( {m - \frac{1}{{\frac{{\left( {\frac{b}{{4m\sqrt { - 6ac} }}} \right)}}{\alpha }\left( {x + \frac{1}{{{\Gamma }\alpha }}} \right)^{\alpha } - \frac{{ - \frac{3}{16am}\left( {\frac{{b^{3} }}{{4\sqrt { - 6ac} }}} \right)}}{\beta }\left( {t + \frac{1}{{{\Gamma }\beta }}} \right)^{\beta } }}} \right)}}. \\ \end{aligned}$$

This methodical arrangement ensures a comprehensive analytical framework for the realized soliton solutions, categorizing their functional expressions based on parameter constraints.

## Impact of stretching exponents

To investigate solitons, the solution further provides conventional data represented graphically as actual solution amplitudes and null solutions. It employs 3D and 2D plots, contour graphs, and amplitude profiles to describe their dynamic behavior, such as frequencies, evolutions of the phases, and dynamics. This characterization in multiple dimensions helps to elucidate soliton dynamics and explain solutions acquired.

The parameters $\alpha$ and $\beta$ , control the spatial and temporal stretching of the wave patterns, respectively. When $\alpha = 1$, we have a uniform wavelength. While $\alpha > 1$, the $\alpha$ distorts waves and shoals the fronts. If $0 < \alpha < 1$, it distorts them, contrasting them and flattening profiles. Similarly, $\beta = 1$ provides uniform frequency. If $\beta > 1$, the frequency of oscillations of the wave increases with time, and $0 < { }\beta < 1$ slows them. Overall, the sharpness with which the wave structures vary in space is determined by $\alpha$ and their apparent speed and evolution in time are controlled by $\beta$. This was analyzed in terms of the qualitative behavior of fractional orders; however, numerical results showed quantitative behavior. As the temporal order $\beta$ is increased, soliton amplitude tends to decrease and width tends to increase, and this variation is attributed to the increased memory effects that redistribute energy over longer time durations. Conversely, a change in the spatial order $\alpha$ affects scaling velocity through the dispersion term; specifically, a smaller alpha results in slower but more localized solitons. Although no closed-form scaling law was obtained, the observed correlations will be presented in numbers and will be incorporated into the modified discussion to further clarify the impact of the fractional orders.

**Fig. 2:**
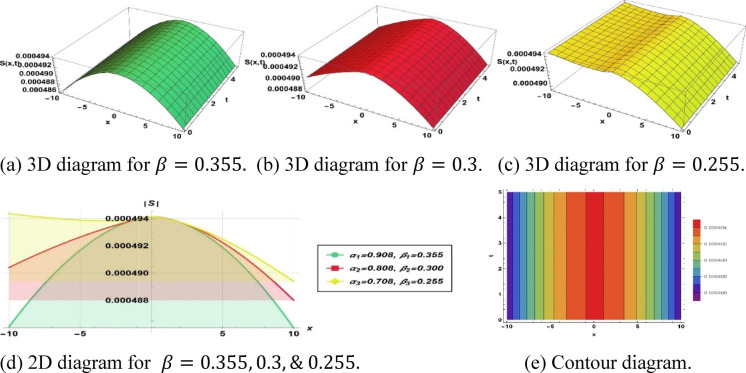
Parametric effect on the concave-shaped soliton to the Eq. (12) for different values.

Figure 2 depicts the solution (12) as a concave-shaped soliton, utilizing the following parameter values $k=0.474, a=0.432, {h}_{1}=0.494, m=1, p=-0.001, {\alpha }_{1}=0.908, {\beta }_{1}=0.355, {\alpha }_{2}=0.808, {\beta }_{2}=0.3$ and ${\alpha }_{3}=0.708, {\beta }_{3}=0.255$.

**Fig. 3:**
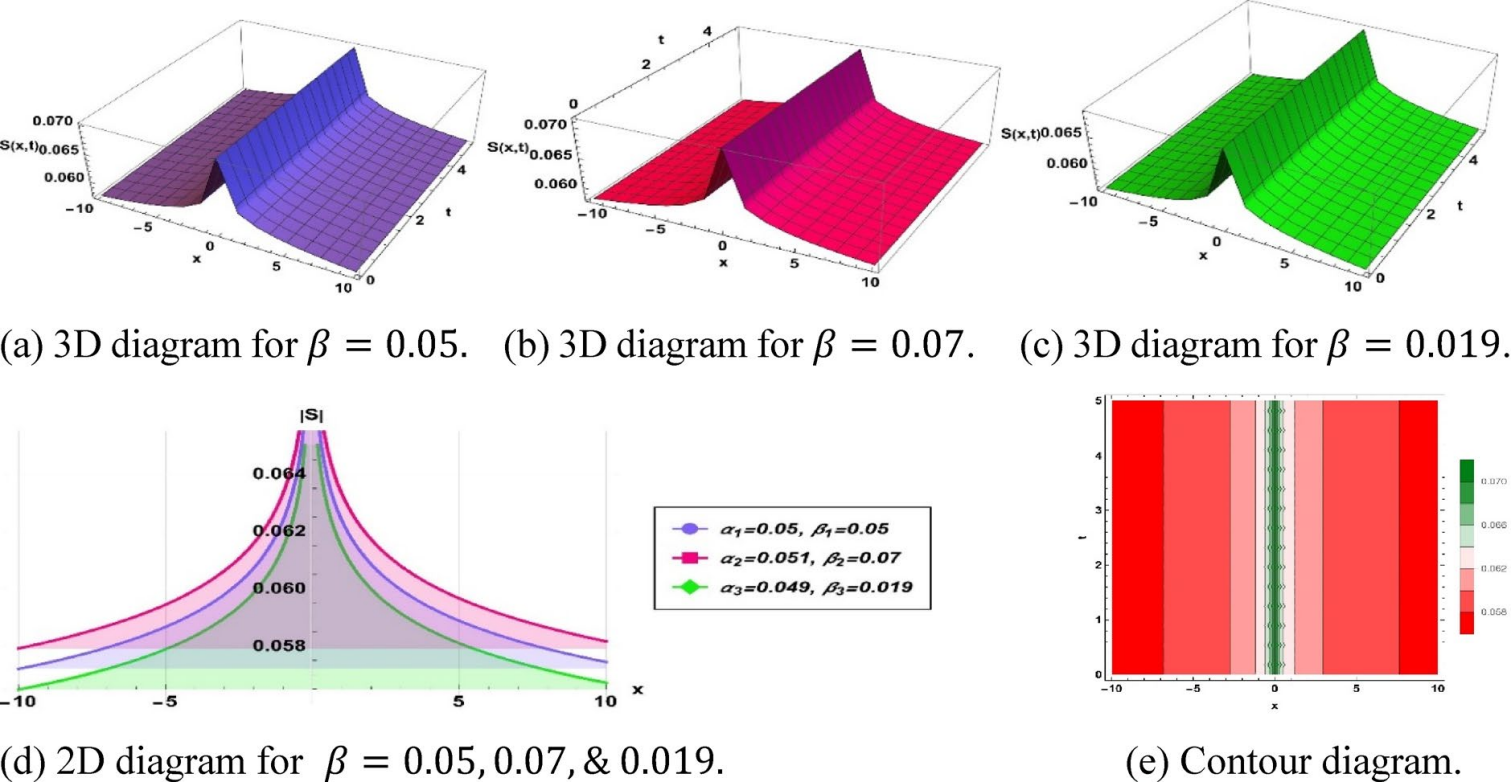
Parametric effect on the single peak type soliton to the Eq. (13) for different values.

The implementation of the specified solution (13) with parameters $k=-0.001, a=-0.01, {h}_{1}=-0.001, m=-10, p=-0.001, {\alpha }_{1}=0.05, {\beta }_{1}=0.05, {\alpha }_{2}=0.051, {\beta }_{2}=0.07$ and ${\alpha }_{3}=0.049, {\beta }_{3}=0.019$ results in a single peak type soliton, as depicted in Fig. 3.

**Fig. 4:**
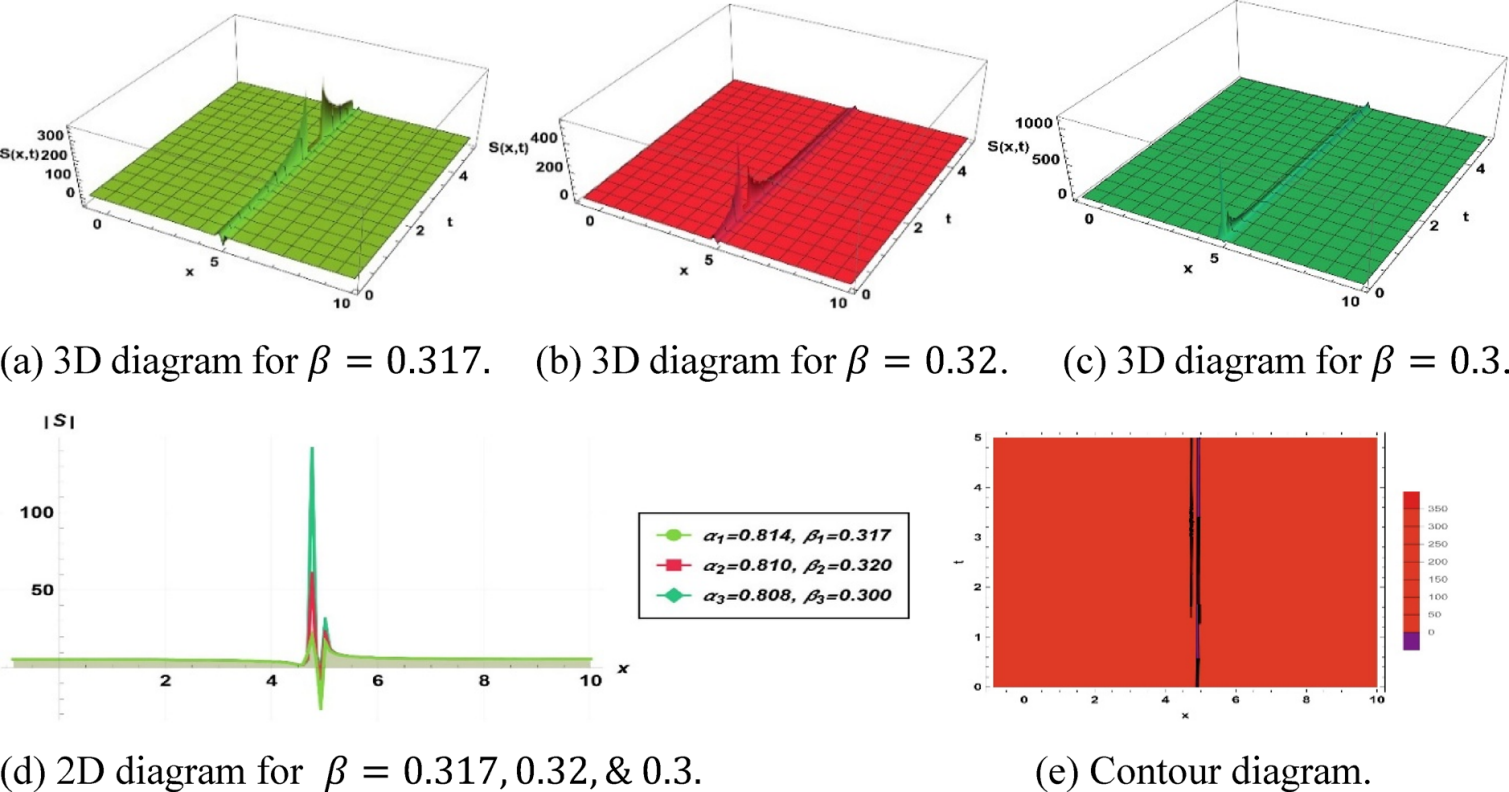
Parametric effect on the bright type of soliton to the Eq. (14) for different values.

Solution (14) produces a bright type soliton, seen in Fig. 4, characterized by the characteristics $k=0.487, a=0.75, {h}_{1}=0.206, m=13.2, p=0.423, {\alpha }_{1}=0.814, {\beta }_{1}=0.317, {\alpha }_{2}=0.810, {\beta }_{2}=0.32$ and ${\alpha }_{3}=0.808, {\beta }_{3}=0.3$.

**Fig. 5:**
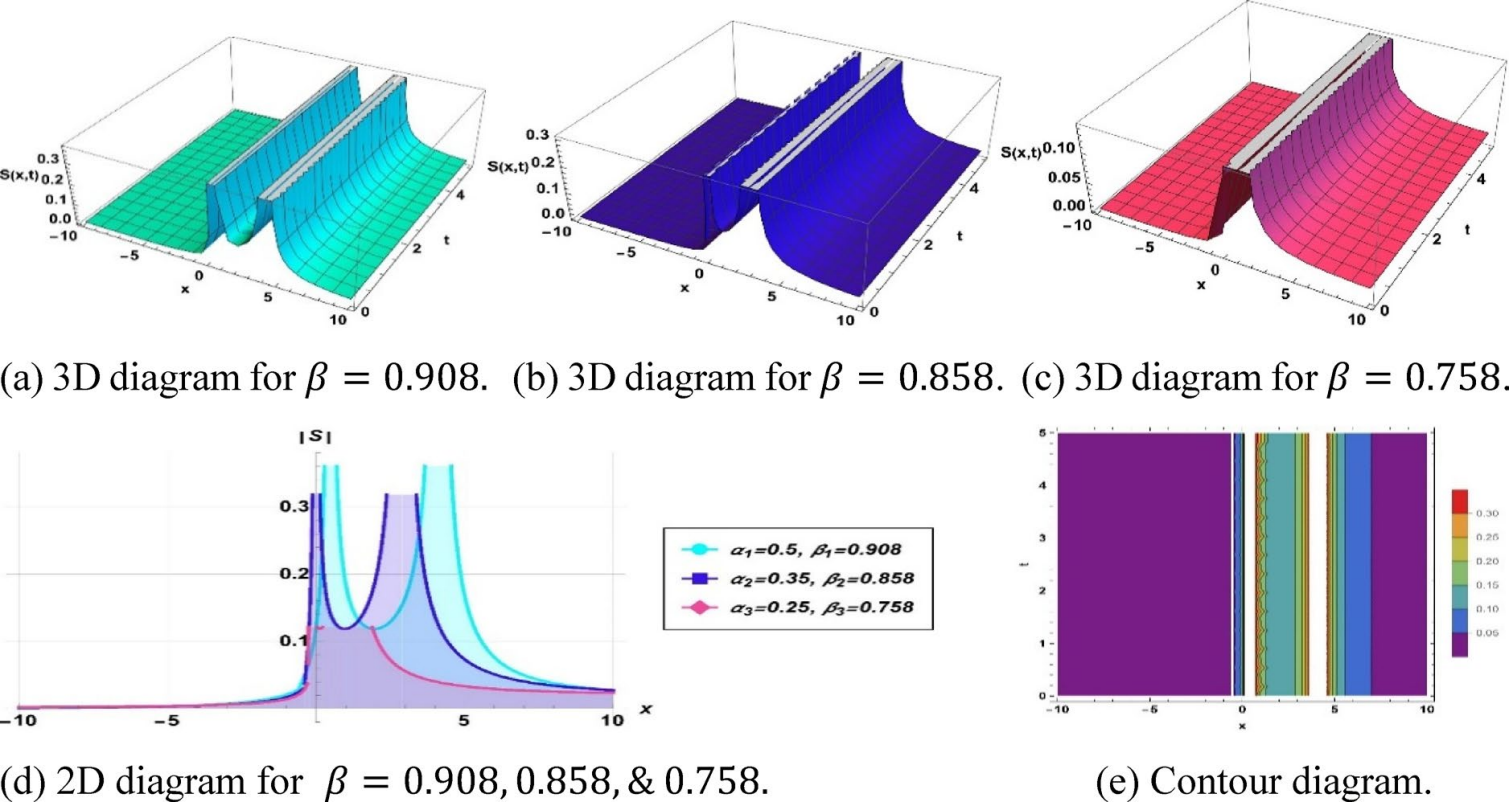
Parametric effect on the shock type soliton to the Eq. (14) for different values.

The specified Eq. (14) depicts a shock type soliton characterized by $k=0.386, a=0.096, {h}_{1}=0.028, m=-0.85, p=0.83, {\alpha }_{1}=0.5, {\beta }_{1}=0.908, {\alpha }_{2}=0.35, {\beta }_{2}=0.858$ and ${\alpha }_{3}=0.25, {\beta }_{3}=0.758$. in Fig. 5.

**Fig. 6:**
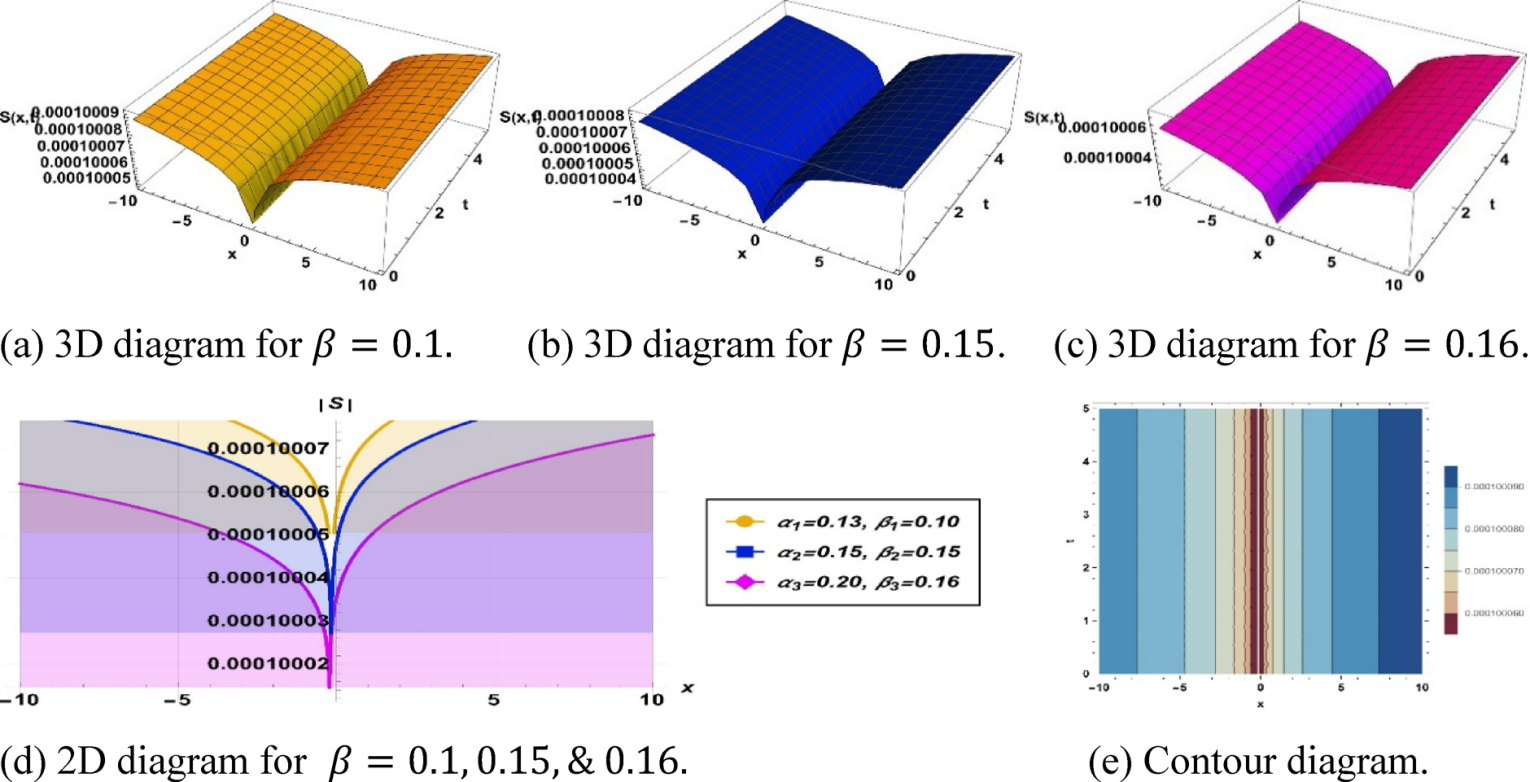
Parametric effect on the dark soliton to the Eq. (12) for different values.

Solution (12) illustrates a dark soliton, as shown in Fig. 6, with the parameters $k=-0.001, a=-0.01, {h}_{1}=-0.001, m=-10, p=-1, {\alpha }_{1}=0.13, {\beta }_{1}=0.1, {\alpha }_{2}=0.15, {\beta }_{2}=0.15$ and ${\alpha }_{3}=0.2, {\beta }_{3}=0.16$.

**Fig. 7:**
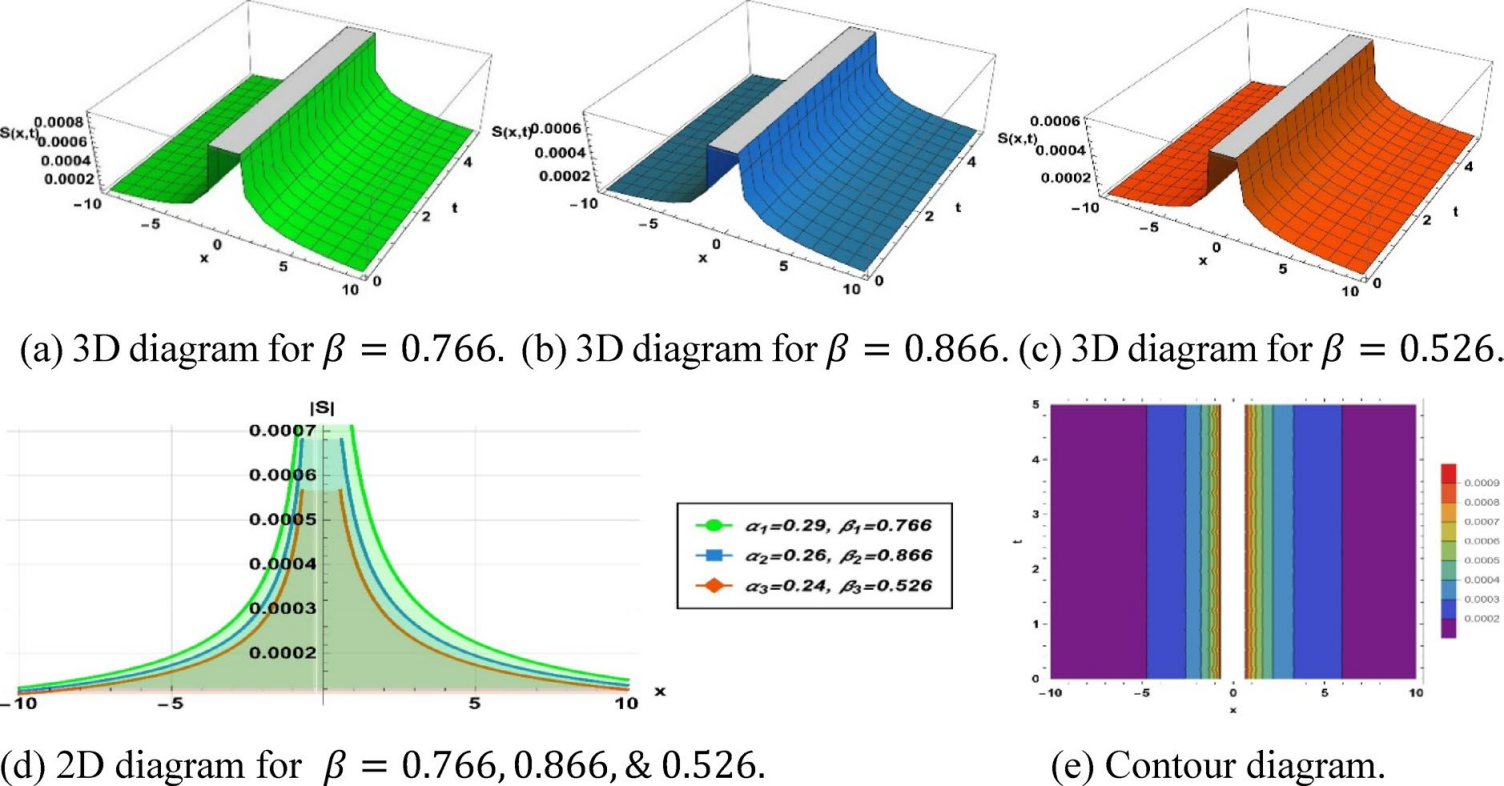
Parametric effect on the pulse type soliton to the Eq. (15) for different values.

Figure 7 illustrates the pulse type soliton derived from solution (15), employing the parameters $k=0.406, a=0.008, {h}_{1}=-0.001, m=1.3, p=0.001, {\alpha }_{1}=0.29, {\beta }_{1}=0.766, {\alpha }_{2}=0.26, {\beta }_{2}=0.866$ and ${\alpha }_{3}=0.24, {\beta }_{3}=0.526$.

**Fig. 8:**
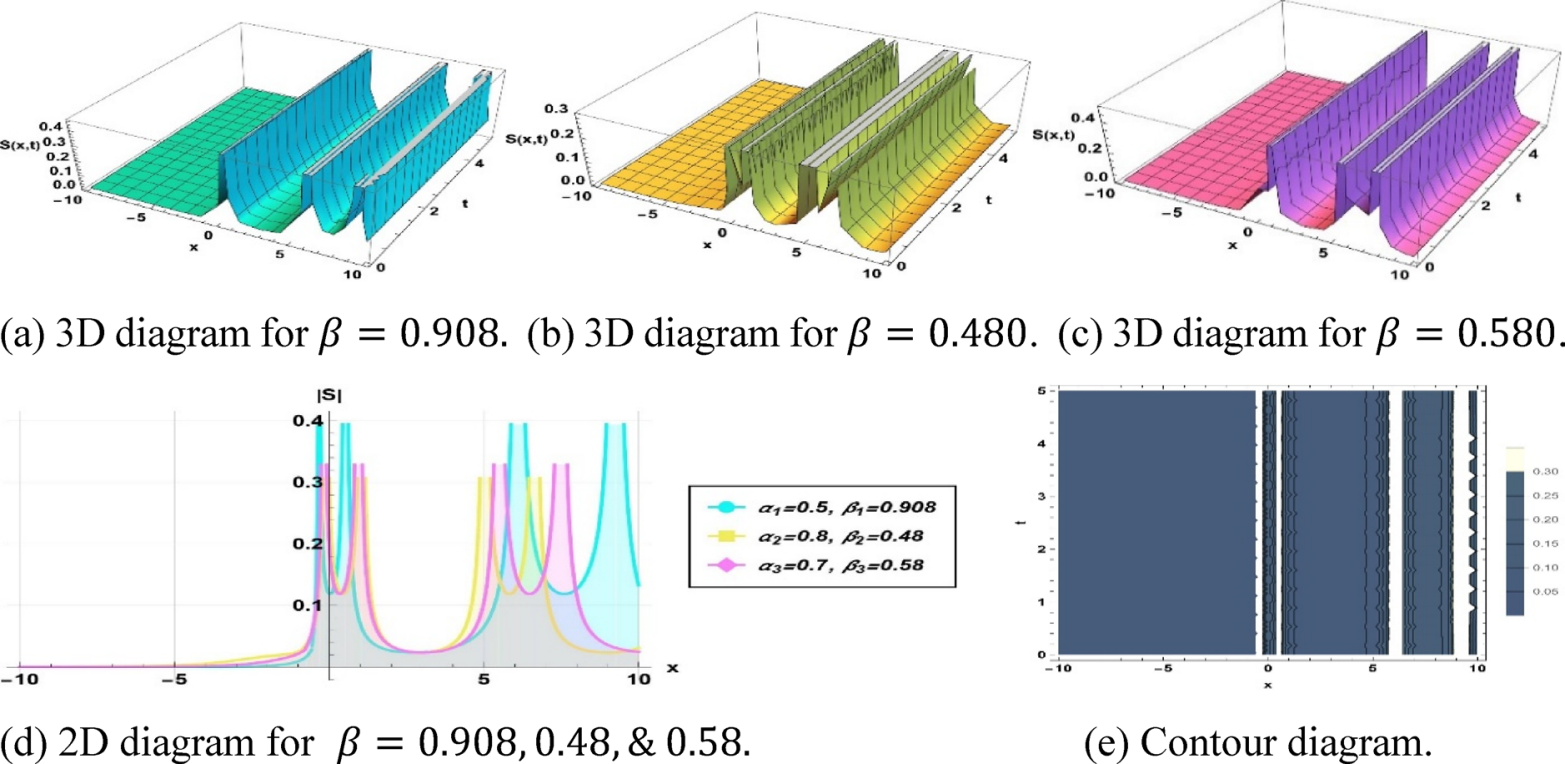
Parametric effect on the periodic soliton to the Eq. (14) for different values.

Figure 8 illustrates a periodic soliton derived from the previously given solution (14), contingent upon the precise parameterization of $k=0.8, a=0.096, {h}_{1}=0.028, m=-0.85, p=0.883, {\alpha }_{1}=0.5, {\beta }_{1}=0.908, {\alpha }_{2}=0.8, {\beta }_{2}=0.48$ and ${\alpha }_{3}=0.7, {\beta }_{3}=0.58$.

**Fig. 9:**
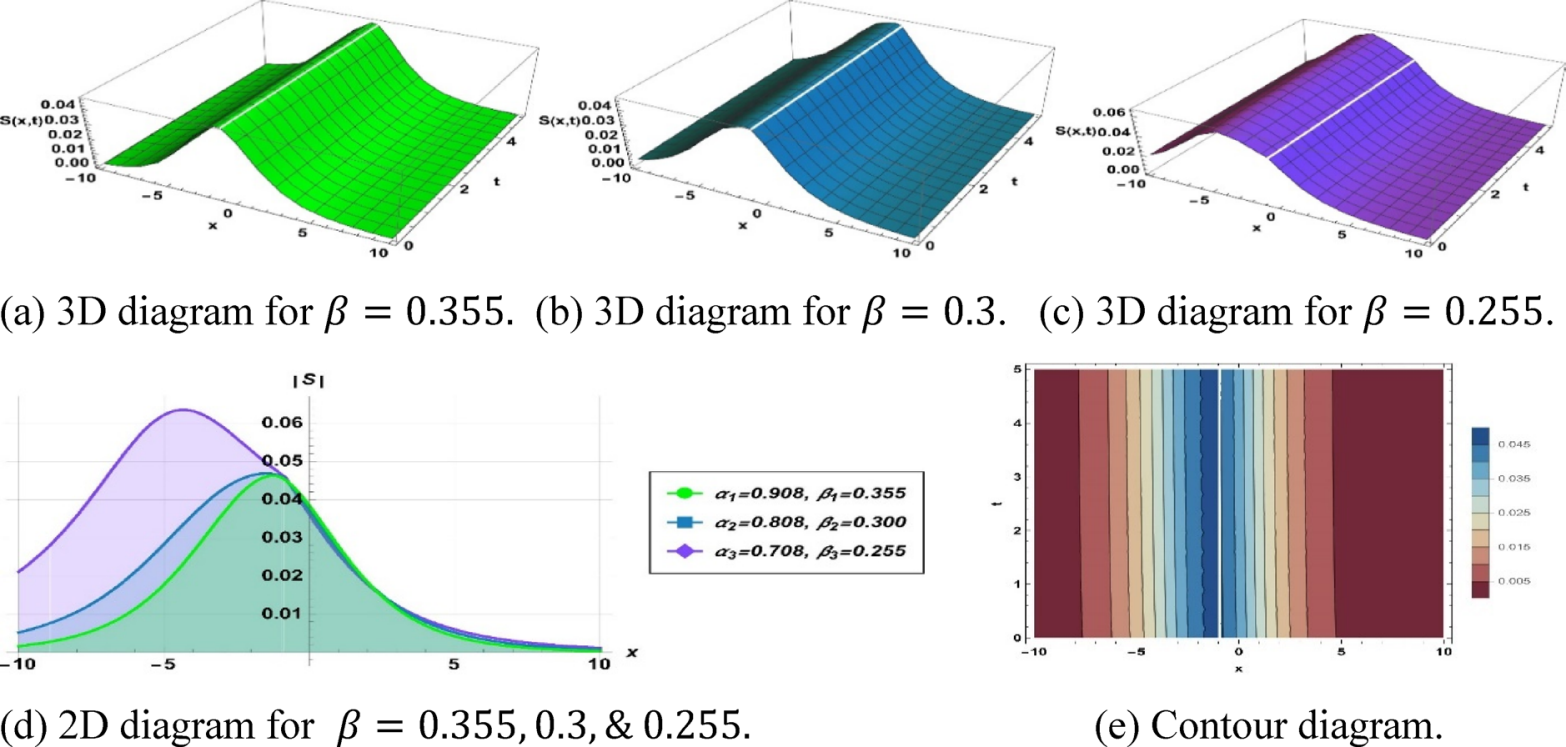
Parametric effect on the kink type soliton to the Eq. (12) for different values.

Modifying the parameters to $k=0.376, a=0.432, {h}_{1}=0.494, m=-7.65, p=-0.713, {\alpha }_{1}=0.908, {\beta }_{1}=0.355, {\alpha }_{2}=0.808, {\beta }_{2}=0.3$ and ${\alpha }_{3}=0.708, {\beta }_{3}=0.255$ induces a significant alteration in the solution (12), yielding a kink type soliton, as depicted in Fig. 9.

The parameter values used for soliton profiles were determined through a combination of analytic admissibility conditions and numerical experimentation. The enhanced F-expansion approach imposes parameter restrictions (e.g., ensuring non-singularity, reality, and nonlinear-dispersive balance) that define admissible ranges for parameters like “*a*”, “*h*_*1*_”, and “*p*”. Within these ranges, numerical exploration identified representative parameter sets yielding qualitatively distinct soliton structures (concave, bright, dark, and kink-type). The specific values exemplified $( a=0.432, {h}_{1}=0.494, p=-0.001)$ were selected as they produce well-resolved and physically interpretable waveforms. It is emphasized that these observed soliton types are not exclusive to these precise parameter values but exist across continuous families of solutions within these regimes, with the chosen values serving to illustrate the variety of nonlinear structures permitted by the fractional mKdV-Burger equation.

## Physical significance of soliton

Figure 2 represents a structure related to a concave soliton solution, i.e., a confined, nonlinear, wave-like structure with a so-called valley-shaped characteristic. Such waves have been defined as depression-type, or localized holes within continuous media. This soliton is essential for simulating negative energy pulses or decreases in density in the system of dissipative dispersion. Additionally, this type of soliton highlights the localized energy redistribution and its potential role in controlling wave damping or instabilities in nonlinear media***.*** Figure 3 depicts a single-peak soliton solution and is characterized by a localized oscillation of a significant crest and even-handed decay on both sides, which is commonly associated with brilliant solitons in optical fibers, solitary surface waves in liquid channels, and constrained plasma oscillations. Furthermore, it demonstrates how the soliton can maintain its shape over long distances, emphasizing stability and robustness in nonlinear wave propagation. Figure 4 shows a bright-type soliton with clear, discrete peaks with coherent shapes and indicates strong energy localization of the soliton within the nonlinear dispersive media. Such formations are commonly associated with rogue waves and intense pulses because of their resistance to dispersion. These solutions are important for understanding extreme events in oceanography and high-intensity pulse dynamics in nonlinear optics. Figure 5 is the so-called kink- or shock-type solitons, which mark a smooth passage between two rest states. These solutions address nonlinear shock propagation, domain wall problems in condensed matter systems, and phase transitions in plasmas. Additionally, they provide insight into wavefront interactions and the evolution of topological structures in nonlinear systems. Figure 6 depicts a dark or small-amplitude soliton that refers to a dip of localized nature within a wave of continuous background and plays an important role in the study of Bose–Einstein condensates, plasma depressions, and nonlinear optical systems. It also helps in analyzing modulational stability and the interaction of small-amplitude disturbances with the background field. Figure 7 illustrates a pulse-like one-dimensional on its own for a confined disturbance that maintains the same structure during propagation, which is a pulse that is achieved by a complex balance between nonlinearity and dispersion. This emphasizes its potential for energy transport in one-dimensional media and the formation of stable coherent structures. Figure 8 illustrates a periodic wave train, like cnoidal oscillations, which represent non-linear periodic oscillations found in fluids, plasmas, and lattice vibrations. These waves are also useful for studying modulational instability, recurrence phenomena, and energy sharing between modes in nonlinear periodic systems. Figure 9 shows a kink or front-type soliton between two different asymptotic stages. Such solutions are critical for modeling front propagation, phase transitions, and boundary layer dynamics in dispersive media.

## Investigation of stability

**Fig. 10:**
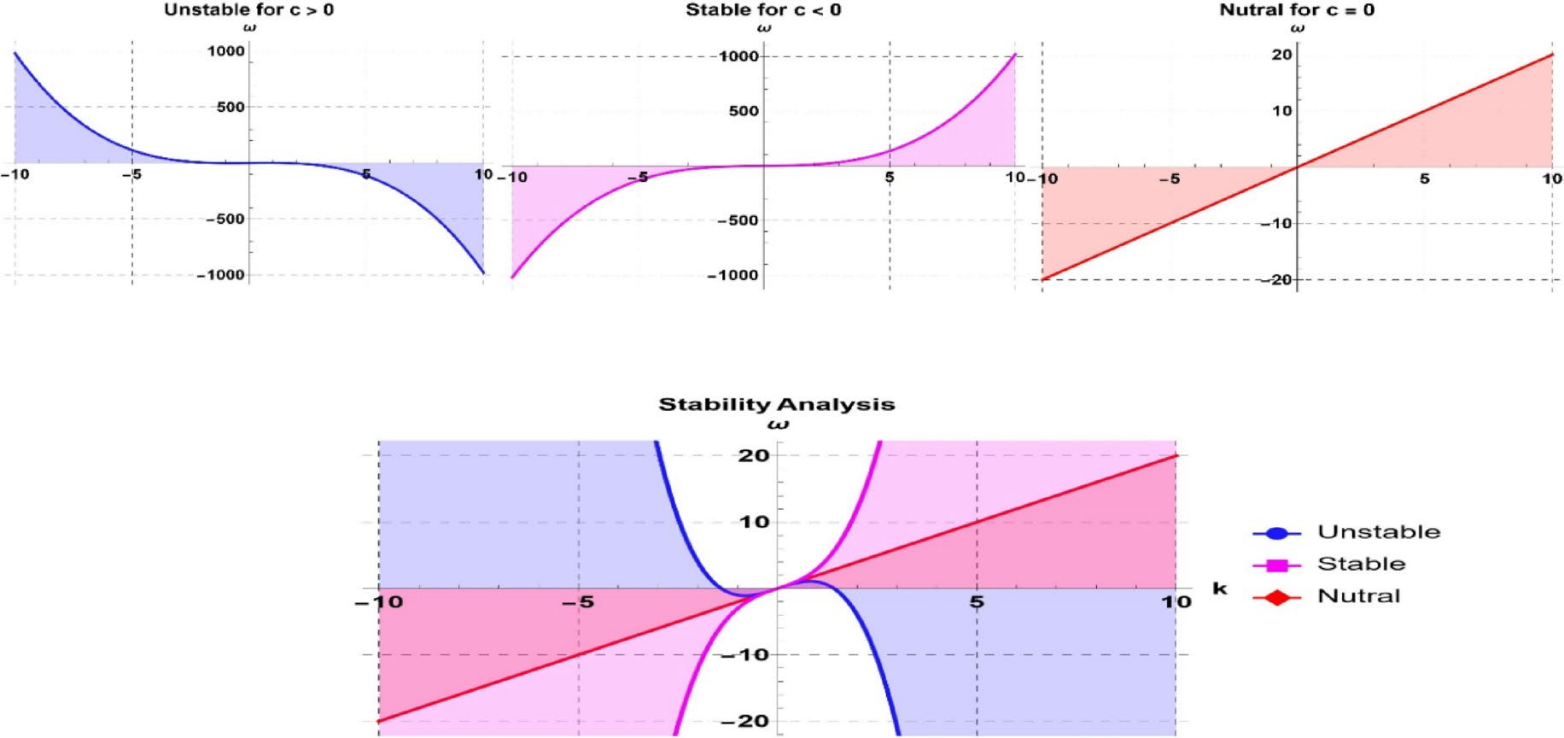
Visualization of stability analysis in 2D patterns to the Eq. (20) for different values.

Stability analysis refers to an algebraic methodology that has been used to try to measure the stability or instability of a system to small variations in time. It classifies operating zones as unstable and stable and makes the arrangement restricted and predictable. The stability of the mKdV-Burger’s Eq. is studied using a complex linear stability method that can be considered more complicated. The procedure specifies how the system would react to small perturbations and identifies the stability region by perturbing the solution of the fundamental Eq. with a wave having a small amplitude and being time independent, as shown in Fig. 10.

17 $$S\left( {x,t} \right) = U_{0} + \varepsilon U\left( {x,t} \right).$$

$U_{0}$ describes a solution to the framework of the equilibrium condition. $\varepsilon$ is a small constant value. $U\left( {x,t} \right)$ is the perturbed solution of the wave. Replacing solution into Eq. (1) and dropping higher-order terms in $\varepsilon$ as well as linearizing the obtained Eq., we get

18 $$D_{t}^{\beta } U + aU_{0}^{2} D_{x}^{\alpha } U + bU_{0} D_{x}^{\alpha } U + cD_{x}^{3\alpha } U = 0.$$

The Eq.is a linear differential Eq. pertaining to the wave variable $U\left( {x,t} \right).$ The proposed solution for Eq. (18) can be articulated as follows:

19 $$U\left( {x,t} \right) = e^{{i\left( {\frac{k}{\alpha }\left( {x + \frac{1}{{{\Gamma }\alpha }}} \right)^{\alpha } - \frac{\omega }{\beta }\left( {t + \frac{1}{{{\Gamma }\beta }}} \right)^{\beta } } \right)}} .$$

In this case, the symbols $k$, the wave number and $\omega$ are used to represent the nonlinear nature or the fractional exponent, and $\omega$ specifies the frequency or the phase velocity of the perturbed wave. By replacing the trial solution (19) in Eq. (18). In the derivation of the dispersion relation, Eq. (17) leads to obtaining the phase velocity $\omega$ as

20 $$\omega = k\left( {aU_{0}^{2} + bU_{0} } \right) - ck^{3} .$$

## Bifurcation

Bifurcation analysis is a technique applied to test the qualitative behavior of solutions to ODEs of the space–time fractional mKdV-Burger’s equation. This means transforming the fractional PDE into a planar dynamic system including nonlinearity, dispersion, and Burgers-type dissipation. This technique can be used to undertake a systematic investigation of equilibrium configurations and their local phase portraits, which contain centers, saddles, and cusp-like critical points^63^. It is effective, especially when used to chart parameter-dependent transition and the ways in which alterations of fractional derivatives and control parameters form the invariant manifolds. The 2D phase space analysis elucidates the multi-stability and heteroclinic/homoclinic orbits as well as initial conditions, giving a rigorous framework in the dynamics of nonlinear dispersive-dissipative waves in fractional media. This approach not only enhances our understanding of complex wave behaviors but also opens avenues for practical applications in engineering and physics. Let $\phi = S\left( {x,t} \right)$ and $\phi^{\prime} = \psi$ then the dynamical system is

21 $$\left\{ {\begin{array}{*{20}c} {\phi^{\prime} = \psi } \\ {\psi^{\prime} = \frac{1}{{ck^{2} }}\left( {\frac{\omega }{k}\phi - \frac{b}{2}\phi^{2} - \frac{a}{3}\phi^{3} } \right)} \\ \end{array} } \right.$$

This system has a Hamiltonian structure as

22 $$H\left( {\phi ,\psi } \right) = \frac{1}{2}\psi^{2} - \frac{1}{{ck^{2} }}\left( {\frac{\omega }{2k}\phi^{2} - \frac{b}{6}\phi^{3} - \frac{a}{12}\phi^{4} } \right).$$

There are three equilibrium points in this system, which are

$$\left( {0,0} \right), \left( {\frac{{ - \frac{3b}{2} + \sqrt {\frac{{9b^{2} }}{4} + \frac{12a\omega }{k}} }}{2a},0} \right),\left( {\frac{{ - \frac{3b}{2} + \sqrt {\frac{{9b^{2} }}{4} + \frac{12a\omega }{k}} }}{2a},0} \right).$$

Moreover, the Jacobian for the system is

$$J\left( {\phi ,\psi } \right) = \left| {\begin{array}{*{20}c} 0 & 1 \\ {\frac{1}{{ck^{2} }}\left( {\frac{\omega }{k} - b\phi - a\phi^{2} } \right)} & 0 \\ \end{array} } \right|.$$

The eigenvalues of the system under the equilibrium point $\left( {0,0} \right)$ are

$$\lambda = \pm \sqrt {\frac{\omega }{{ck^{3} }}} .$$

**Fig. 11:**
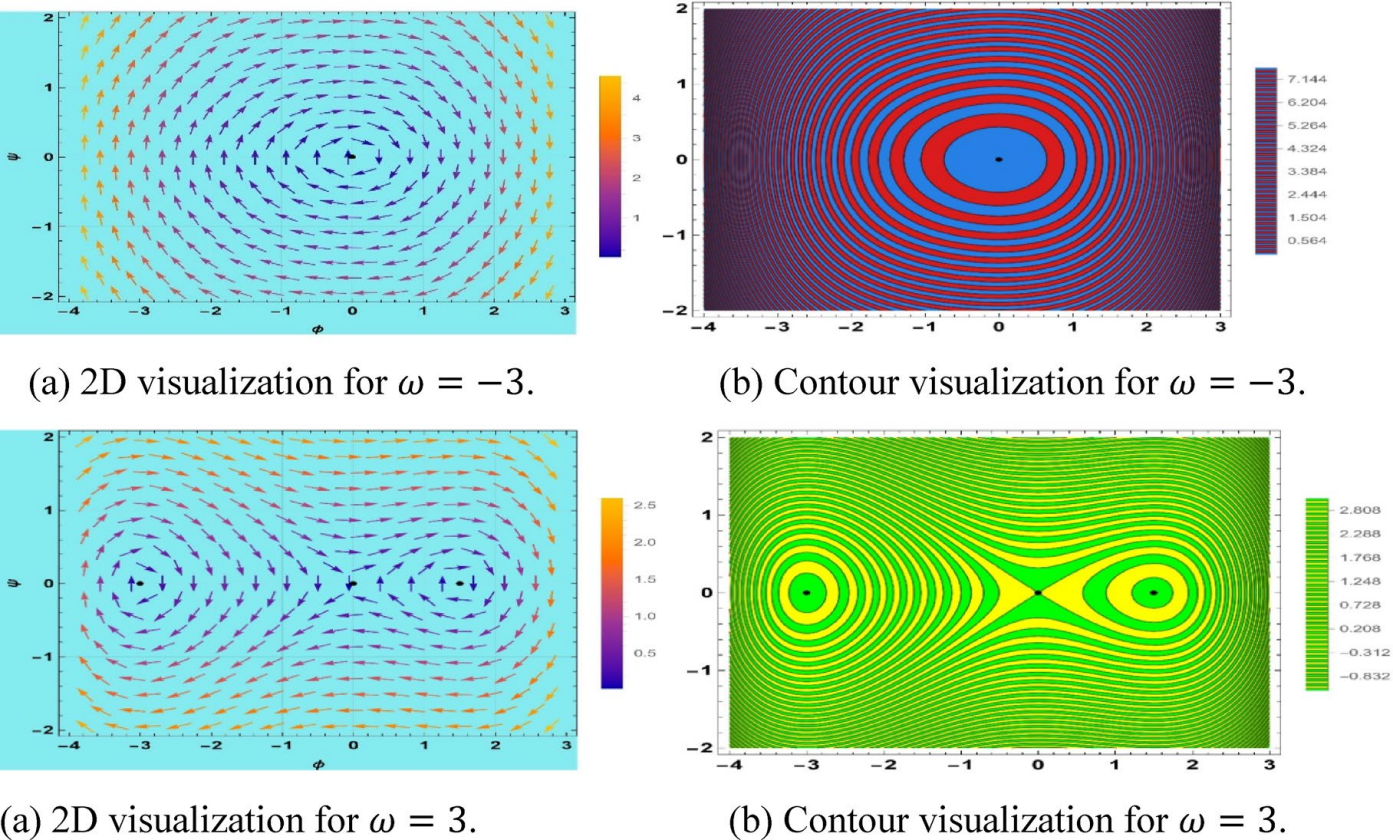
Visualization of supercritical bifurcation of system (21).

The mKdV-Burgers Eq. provides pitchfork bifurcation. A pitchfork bifurcation is an effect in which the equilibrium points of a system switch to a qualitatively different stable or unstable point whenever a parameter passes a critical value. These bifurcations are classified into two broad classifications: supercritical and subcritical. In supercritical bifurcations, a parameter crosses a critical point, and a stable center at $\left( {0,0} \right)$ is replaced by two unstable equilibrium points at $\left( { \pm \sqrt \omega ,0} \right)$ and $\left( {0,0} \right)$, as illustrated in Fig. 11.

**Fig. 12:**
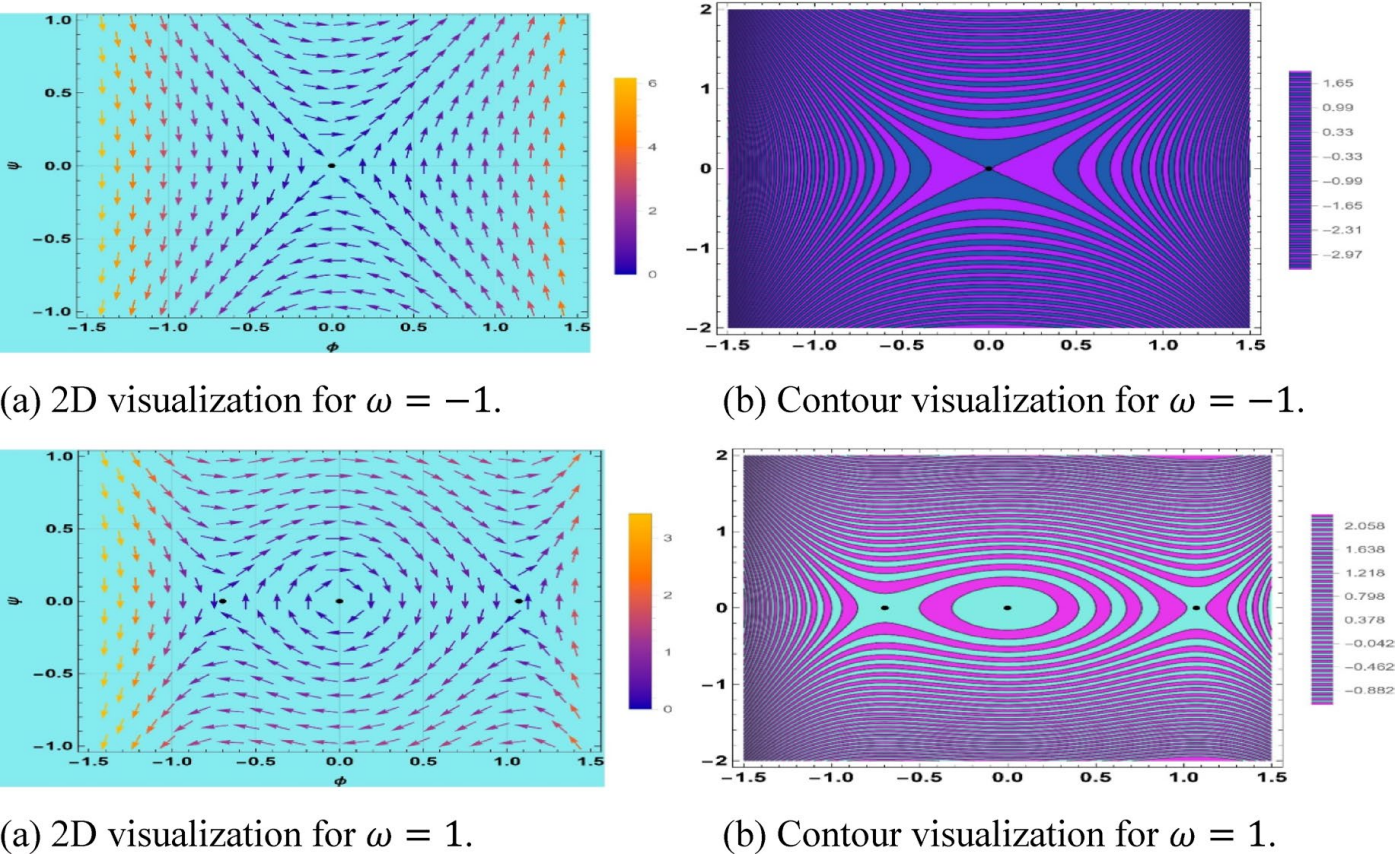
Visualization of subcritical bifurcation of system (21).

Subcritical bifurcations are the ones where the initial equilibrium could remain stable, then it was shifted into an unstable one, which is a dissimilar course. The stable solutions of the interested system are at the origin of $\left(\text{0,0}\right)$, whereas the equilibrium solutions are always unstable, as demonstrated in Fig. 12.

In bifurcation analysis, the driving frequency $\omega$ is identified as the primary bifurcation parameter, directly influencing qualitative shifts in the periodic, quasi-periodic, and chaotic dynamics of the reduced dynamical system. The wave number $k$, while a scaling parameter that modifies dispersion strength, does not function as a control variable for generating bifurcation branches. As clarified in this section, $\omega$ serves as the control parameter in bifurcation diagrams, with other parameters held constant to highlight the bifurcation structure.

## Chaotic nature

Chaotic systems are highly sensitive to the initial condition^63^: small changes in the initial state are amplified, and long-term behavior can thus very well become considerably different. It is found elsewhere in nature and artificial systems, such as fluids, optical systems, atmospheric processes, and so-called organ-on-a-chip devices. This study shows the chaotic nature of the space–time fractional mKdV-Burgers equation, considering the effect of changes in the frequency and amplitude parameters within the system. Given in Eq. (21), the following coupled differential Eqs are obtained in the case of the current system:

23 $$\left\{ {\begin{array}{*{20}c} {\phi^{\prime} = \psi } \\ {\psi^{\prime} = \frac{1}{{ck^{2} }}\left( {\frac{\omega }{k}\phi - \frac{{b\phi^{2} }}{2} - \frac{{a\phi^{3} }}{3}} \right) + \gamma sin\left( {\eta t} \right)} \\ \end{array} } \right.$$

**Fig. 13:**
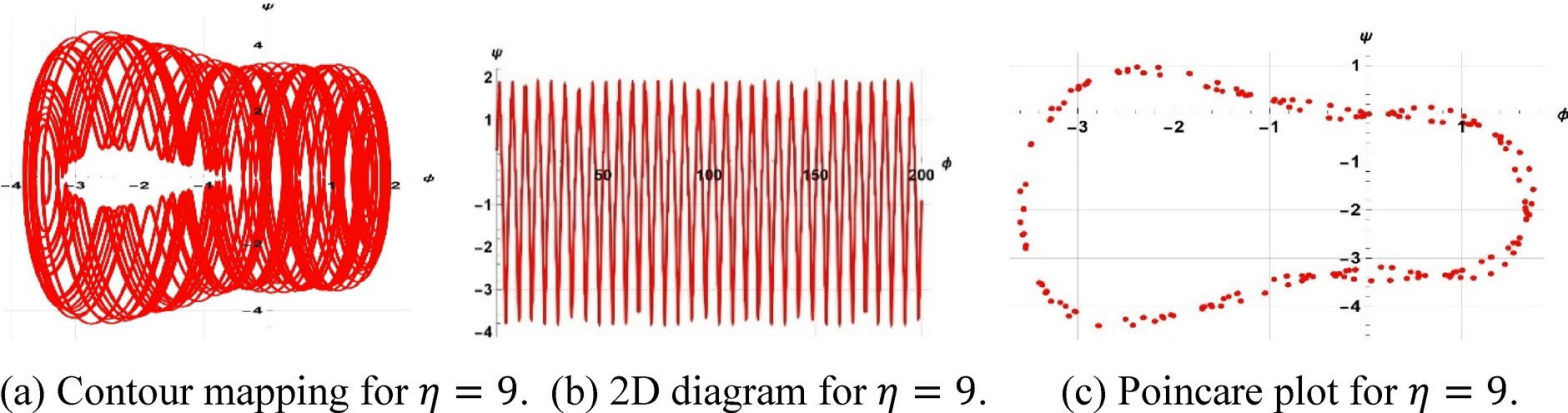
Depiction of periodic behaviors of system (23).

**Fig. 14:**
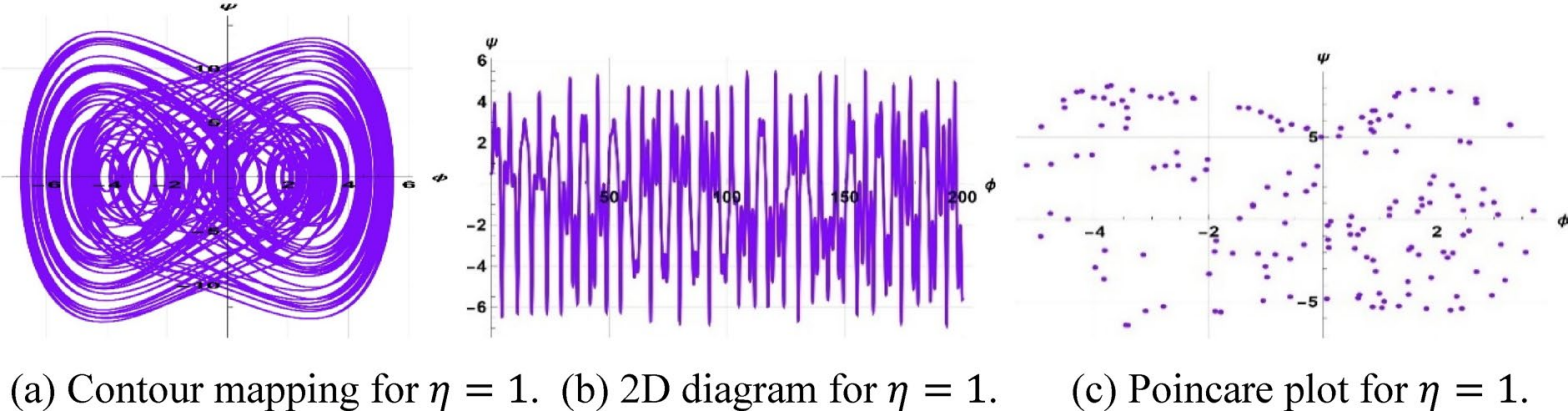
Depiction of chaotic behaviors of system (23).

**Fig. 15:**
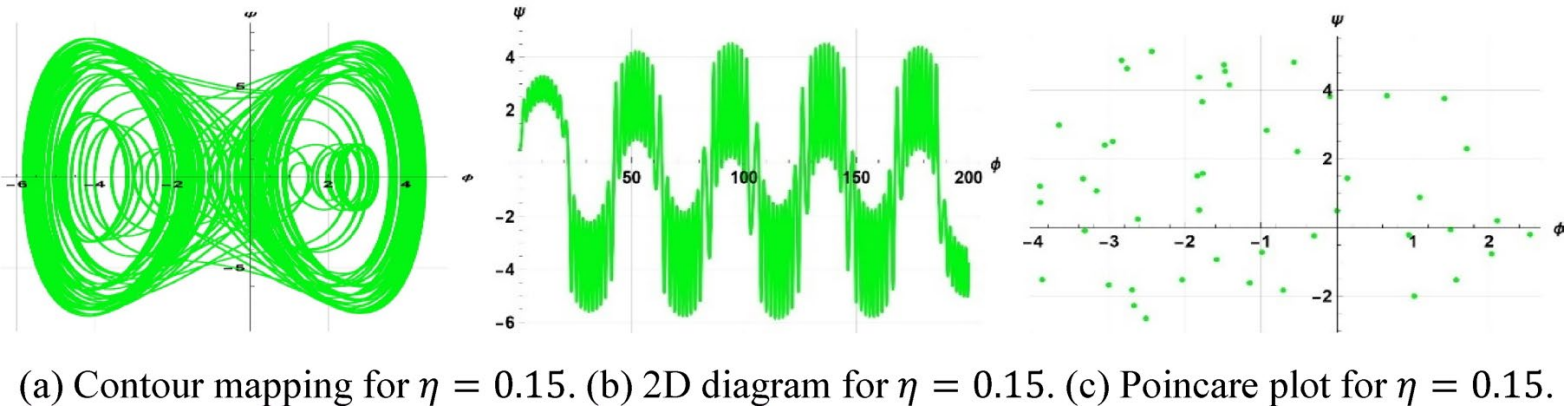
Depiction of quasi-periodic behaviors of system (23).

The system considered is that described by Figs. 13, 14, and 15 are respective examples of Eq. (23) effects on the perturbation term $\gamma sin(\eta t)$. The symbol $\gamma$ that appears here represents the amplitude of the external driving term, and the character $\eta$ represents the frequency of the periodic perturbation. We presently consider the case when the system is typified by Eq. (23) depends on the mentioned values of frequency and amplitude. The investigation analyzes a system affected by frequency and strength factors, uncovering various frequencies and intensities of chaotic traits. The initial configurations $\left(\text{0.3,0}\right),\left(\text{0.5,0.2}\right), \& (\text{0.5,0})$ exhibit periodic patterns for $\eta =9$, chaotic characteristics for $\eta =1$, and quasi-periodic qualities for $\eta =0.15$, with parameters $k=1, \omega =2,\gamma =15, a=4, b=5, and c=3$ applied.

A two-tier diagnostic process was accomplished to distinguish periodic, quasi-periodic, and chaotic regimes. At first, qualitative approaches such as phase portraits, Poincaré sections, and time series analysis were used to distinguish between regular (periodic, quasi-periodic) and irregular (chaotic) behaviors as the driving frequency varied. These graphical techniques effectively revealed routes to chaos and attractor structures. The largest Lyapunov exponent (LLE) was also calculated on representative trajectories to complement the visual examination. The appearance of chaos was verified by a positive LLE and zero and negative values by the initial conditions of quasi-periodic and periodic behavior, respectively, which provided a numerical foundation of the classification. To illustrate, in the case of the trajectory of Fig. 14 ($\eta =1$), the largest Lyapunov exponent was numerically determined as $\lambda =4.074>0$, which confirms that it is chaotic. Conversely, Fig. 13 ($\eta =9$) obtained $\lambda = -0.408$, which is believed to be in a stable periodic orbit, whereas Fig. 15 ($\eta =0.15$) obtained $\lambda = 0.594$, which can be said to be in a quasi-periodic orbit because it is nearer to zero.

## Multi-stability

**Fig. 16:**
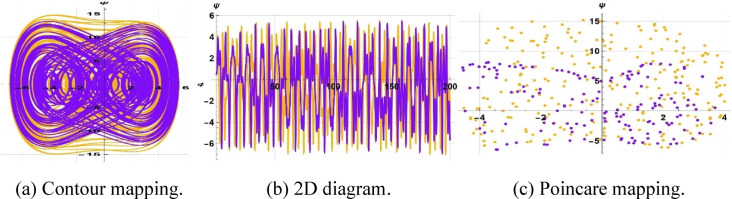
Representation of multi-stability of (21) for $\eta =1 \& \eta =1.5$.

Multi-stability analysis is a technique that determines how uncertain or variable the input parameter is in the results of mathematical models. It can be helpful in detecting factors affecting model productivity and propagating input variability. Such a strategy can be used to determine the strength, reliability, and stability of computational predictions; therefore, it can be used to make positive decisions within complex systems. The sensitivity to initial conditions is characterized by research using the changed shapes and proving that the periodicity of output is very sensitive to the initial state of the subject system, especially when there are minimal perturbations. The paper supports the importance of the magnitude of the disturbance in determining the stability of the system and dynamic responses. Figure 16 shows the initial configuration $\left(\text{0.5,0.2}\right) \& (\text{0.5,0.3})$ and parameter settings of $k=1, \omega =2, a=4, b=5, c=3, \gamma =15$ and $\eta =1 \& \eta =1.5$ respectively.

**Fig. 17:**
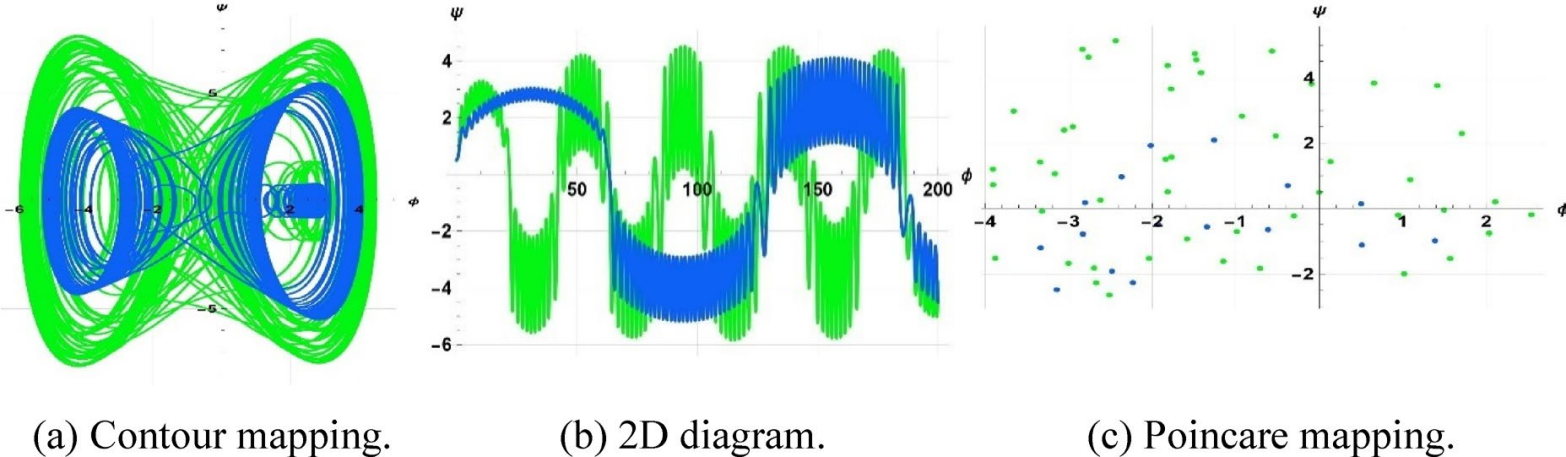
Representation of multi-stability of (21) for $\eta =0.15 \& \eta =.05$.

**Fig. 18:**
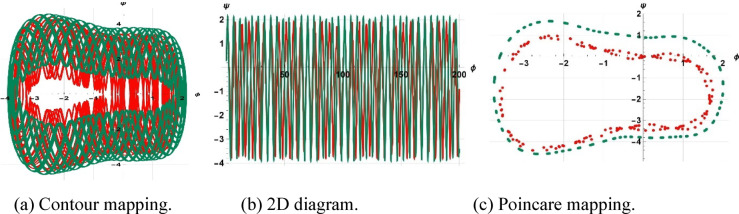
Representation of multi-stability of (21) for $\eta =9 \& \eta =10.5$.

The starting setup $\left(\text{0.5,0}\right) \& (\text{0.5,0.15})$, with parameter values $k=1, \omega =2, a=4, b=5, c=3, \gamma =15$ and $\eta =0.15 \& \eta =0.05$ , is illustrated in Fig. 17. Additionally, the starting configuration $\left(\text{0.3,0.0}\right) \& (\text{0.3,0.9})$, with parameter values $k=1, \omega =2, a=4, b=5, c=3, \gamma =15$ and $\eta =9 \& \eta =10.5$, is illustrated in Fig. 18.

The fractional mKdV-Burgers system displays multi-stability, meaning it is sensitive to initial conditions and can settle into various attractors like periodic, quasi-periodic, or chaotic states, even with unchanged parameters. This behavior of nonlinear wave propagation indicates that even small initial deviations may produce very different long-term dynamics, e.g., a stable soliton or chaotic oscillation. This is worsened by dispersive-dissipative media, which point to the need to control initial excitations in such fields as plasma waves, fluid turbulence, and optical communications. Multi-stability also has the potential of encoding information and switching because small changes in input can be used to cause big changes in output.

## Lyapunov stability

**Fig. 19:**
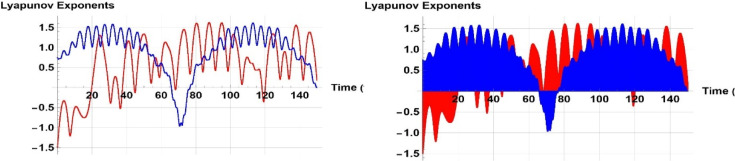
Graphical presentation of Lyapunov stability in 2D patterns of (21).

Figure 19 shows trajectories with negative and positive Lyapunov exponents, indicating chaotic systems with sensitivity to initial conditions. Negative Lyapunov exponents indicate asymptotic stability, while positive exponents indicate sensitivity to beginning circumstances, indicating systems with unusual attractors or aperiodic long-term behavior. These systems have stable fixed points, periodic attractors, or limit cycles, while their predictability is compromised due to their deterministic nature.

## Perspective research

The present paper explores the space–time fractional mKdV-Burgers Eq. through Lyapunov exponents, linear and nonlinear instabilities, bifurcation, and chaos identification strategies. It gives a structure to the diagnosis of instability onset, characterization of transition to chaos, and sensitivity of initial conditions. The method predisposes more refined modeling and experimental checks of dissipative dispersive solitary waves in the conditions of fractional-order consequences. The results would have important contributions to the study of nonlinear wave propagation, fluid dynamics, and modeling of turbulence, as well as the study of plasmas, straddling the mathematical formalism to practical applications in broad science and engineering fields. Avoiding the need to consider a particular physical system, the space–time fractional mKdV-Burgers Eq. is an appropriate model in systems where nonlinear convection, dispersion, dissipation, and memory all dominate. Examples include simple nonlinear wave steepening in dusty plasmas and collisional dissipation, bright and dark and kink-type solitons in optical fibers and nonlocal or gain–loss effects, and porous or viscoelastic beds, permitting thin flows of water with a fractional dissipation. These illustrate that the families of solitons that we have obtained are not merely mathematically new but can also be experimentally accessible in dispersive-dissipative systems with hereditary interactions.

## Evaluation of novelty

This paper uses the improved F-expansion approach to study nonlinear wave theory, fluid mechanics, plasma dynamics, and applications to media with dissipation and memory effects. The approach enables the retrieval of a wide range of violated solutions, including traveling low-energy waves, hyperbolic, trigonometric, and rational traveling wave solutions. This study presents a unique method, revealing novel soliton solutions that have never been solved before. The study also includes a full dynamical analysis of stability, bifurcation, chaos, multi-stability, sensitivity, and Lyapunov exponents. The findings provide new perspectives on the use of fractional-order effects and nonlinear dissipation in complex dynamics, highlighting the originality of the study. Comparing this method with other literature highlights its value in these fields, as summarized in Table 1.

**Table 1:** Comparative analysis of analytical methods and results for fractional NLEEs.

Study	Framework	Applied strategies	Acquired outcomes
Mustafa et all^64^	Space–time fractional mBBM (and Duffing**)**	Extended tanh function (conformable derivative)	Bell-type, anti-bell, kink, anti-kink, compacton, singular, multi-soliton
Hafiz Uddin et al^65^	Space–time fractional modified BBM, ZKBBM	$(G{\prime}/G, 1/G)$ expansion method	Hyperbolic, trigonometric, and rational solitary wave solutions
Jan Muhammad et al^66^	Space–time fractional mKdV-KPE	Enhanced modified extended tanh-expansion method	Solitary waves: qualitative parametric analysis
Current investigations	Space–time fractional mKdV–Burger’s equation	Modified F-expansion Function Method	Several solitons solutions, Multi-stability, Lyapunov exponents stability & bifurcation, and chaos behavior graphical representation

We assert our work is novel due to the integration of an enhanced F-expansion method, which produced unconventional soliton shapes (concave, single-peak), the inclusion of the Burgers dissipation term leading to shock-like structures and dissipative solitons, and the incorporation of dynamical analysis elements such as bifurcation diagrams, Lyapunov exponents, sensitivity analysis, and multi-stability exploration. These combined contributions provide a more thorough understanding of the fractional mKdV-Burgers equation, highlighting its mathematical innovation and physical significance.

## Conclusion

In this study, the space–time fractional modified Korteweg-de Vries Burgers (mKdV-Burgers) equation was systematically studied in this work by using the improved method of F-expansion with the incorporated Riccati equation. Such a framework allowed the derivation of a broad variety of precise solutions, such as solitary, periodic, rational, dissipative, and shock-like solitons, far broadening the existing solution space of fractional nonlinear evolution equations (FNLEEs). Detailed dynamical studies, including bifurcation diagrams, phase portraits, Lyapunov exponents, and sensitivity measures, demonstrated the nonlinear dynamics and bifurcations between order and chaos, showing the influence of the fractional parameters on the stability and the dynamics of systems. These findings prove the efficiency and the generality of the improved F-expansion technique and highlight the physical significance of fractional derivatives in describing the effects of memory and nonlocality. In addition to the theoretical contributions, the results are of practical interest in fluid dynamics, plasma physics, and nonlinear optical systems. It also forms the basis to extend it in the future, such as higher-dimensional models, coupled systems, stochastic perturbations, and experimental validation, which offers a framework of understanding and manipulating complex fractional nonlinear waves.

## Data Availability

The data used to support the findings of this study are included within the article.
